# Feasibility and Acceptability of a Culturally- and Linguistically-Adapted Smoking Cessation Text Messaging Intervention for Latino Smokers

**DOI:** 10.3389/fpubh.2020.00269

**Published:** 2020-06-30

**Authors:** Francisco Cartujano-Barrera, Lisa Sanderson Cox, Evelyn Arana-Chicas, Mariana Ramírez, Jaime Perales-Puchalt, Pamela Valera, Francisco J. Díaz, Delwyn Catley, Edward F. Ellerbeck, Ana Paula Cupertino

**Affiliations:** ^1^Department of Cancer Prevention and Control, Hackensack University Medical Center, Hackensack, NJ, United States; ^2^Department of Population Health, University of Kansas Medical Center, Kansas City, KS, United States; ^3^Department of Urban-Global Public Health, Rutgers University, Newark, NJ, United States; ^4^Department of Biostatistics, University of Kansas Medical Center, Kansas City, KS, United States; ^5^Center for Children's Healthy Lifestyles & Nutrition, Children's Mercy Kansas City, Kansas City, MO, United States; ^6^James P. Wilmot Cancer Institute, University of Rochester Medical Center, Rochester, NY, United States

**Keywords:** Latinos, m-health, smoking, smoking cessation, text messages

## Abstract

**Objective:** Assess the feasibility and acceptability of a culturally- and linguistically-adapted smoking cessation text messaging intervention for Latino smokers.

**Methods:** Using a community-based strategy, 50 Latino smokers were recruited to participate in a smoking cessation pilot study. Participants received a 12-week text messaging intervention and were offered Nicotine Replacement Therapy (NRT) at no cost. We assessed biochemically verified abstinence at 12 weeks, text messaging interactivity with the program, NRT utilization, self-efficacy, therapeutic alliance, and satisfaction.

**Results:** Participants were 44.8 years old on average (SD 9.80), and they were primarily male (66%) and had no health insurance (78%). Most of the participants were born in Mexico (82%) and were light smokers (1–10 CPD) (68%). All participants requested the first order of NRT, and 66% requested a refill. Participants sent an average of 39.7 text messages during the 12-week intervention (SD 82.70). At 12 weeks, 30% of participants were biochemically verified abstinent (88% follow-up rate) and working alliance mean value was 79.2 (SD 9.04). Self-efficacy mean score increased from 33.98 (SD 10.36) at baseline to 40.05 (SD 17.65) at follow-up (*p* = 0.04). The majority of participants (90.9%, 40/44) reported being very or extremely satisfied with the program.

**Conclusion:** A culturally- and linguistically-adapted smoking cessation text messaging intervention for Latinos offers a promising strategy to increase the use of NRT, generated high satisfaction and frequent interactivity, significantly increased self-efficacy, produced high therapeutic alliance, and resulted in noteworthy cessation rates at the end of treatment. Additional testing as a formal randomized clinical trial is warranted.

## Introduction

Tobacco use remains the leading preventable cause of disease and death among Latinos (1), the largest minority group in the United States (U.S.) (2). Of the ~60 million Latinos than reside in the U.S. (2), 9.8% are current cigarette smokers (3). Compared to both African Americans and non-Hispanic Whites, Latinos are less likely to receive advice to quit and utilize smoking cessation pharmacotherapy and counseling (4–9). According to the 2015 National Health Interview Survey, only 16.6% of Latinos have used cessation pharmacotherapy compared to 32.6% of non-Hispanic Whites (10). Use of counseling services to quit smoking is also lower in this group (5.1 vs. 6.9%) (10).

A myriad of factors account for low utilization of smoking cessation treatment among Latinos. Overall, Latinos report a lack of knowledge about smoking cessation treatment options and perceive a lack of cultural sensitivity when smoking cessation resources are available in Spanish (11). Moreover, Latinos hold several misconceptions about smoking dependence and cessation and may tend to avoid pharmacotherapy, viewing smoking as a weakness rather than an addiction (12, 13). Moreover, non-adherence to smoking cessation pharmacotherapy has been identified as an important barrier to smoking cessation success among Latino smokers (14, 15). Despite these tobacco-related disparities, there are few effective smoking cessation interventions designed for Latino smokers (16), especially interventions that have the potential for high reach across the Latino community. Advancing treatment for Latino smokers demands accessible, effective, and culturally sound interventions.

The literature demonstrates that text messaging-based smoking cessation interventions are effective (17–22). Text messaging interventions have shown to increase self-efficacy and coping mechanisms by including content related to self-awareness, self-monitoring, and behavioral skill learning (22). Moreover, technology advancements allow for delivery of tailored messages based on individual motivational and behavioral needs to support smoking cessation (17). The effectiveness of smoking cessation text messaging interventions may be even greater among hard-to-reach, socioeconomically disadvantaged, and uninsured populations (18), such as Latinos. However, implementation of text messaging interventions among Latinos remains low despite the fact that Latinos are the highest users of text messages (23). Capitalizing on Latinos' fast adoption of mobile technology, an intervention that fosters engagement and trust through cultural congruency and addresses both pharmacotherapy use and behavior change skills to promote smoking cessation could dramatically reduce tobacco-related disparities. Cultural and linguistic congruence may increase the effectiveness of interventions by enhancing message processing and acceptance by participants (24, 25). The purpose of this pilot study was to assess the feasibility and acceptability of a culturally- and linguistically-adapted smoking cessation text messaging intervention for Latino smokers.

## Methods

### Study Design

This is a single-arm pilot study with 50 Latino smokers who received a culturally- and linguistically-adapted smoking cessation text messaging intervention and Nicotine Replacement Therapy (e.g., nicotine patches, gum, and lozenges; NRT). The study was based at JUNTOS Center for Advancing Latino Health, an academic and community partnership at the University of Kansas Medical Center (KUMC). Study procedures were approved and monitored by the KUMC Institutional Review Board (#STUDY00002725). Participants were compensated a $30 retail store gift card both at baseline and follow-up for their time and transportation.

### Recruitment

Latino smokers were recruited by *Promotores de Salud* (Spanish for Community Health Workers) using community-based recruitment strategies (e.g., conducting presentations about the study, distributing program flyers and educational materials on smoking hazards, etc.) in venues with a high concentration of Latinos (e.g., Latino grocery stores, safety-net clinics, community centers, and churches) and word of mouth from participants and community partners. Recruitment started in March 2016 and ended in June 2016. Final 12-week follow-up assessment was completed in September 2016.

### Eligibility

Individuals were eligible if they (1) self-identified as Hispanic or Latino, (2) were ≥21 years of age, (3) smoked a minimum of 3 days in the past week, (4) had a cellphone with unlimited text messaging capability, and (5) were willing to complete a baseline and 12-week follow-up assessment. Individuals were not eligible if they (1) were currently enrolled in a smoking cessation program or using smoking cessation medication, (2) had an additional household member participating in this study, (3) consumed other forms of tobacco (including e-cigarettes), (4) were currently pregnant, breast-feeding or planning to do so in the next year, or (5) were planning to move from their residential city in the following 6 months.

### Screening and Consent

Bilingual (English and Spanish) trained research staff determined participant eligibility. Individuals who were eligible to participate in the study were scheduled an in-person appointment by research staff. During the in-person appointment, staff discussed all aspects of study participation and confidentiality, answered any questions, and guided eligible smokers through the process of written informed consent. Eligibility assessment and consent were available in the participant's language of preference.

### Intervention

*Kick Buts* is an American adaptation of *Txt2stop*, a smoking cessation text messaging intervention that demonstrated a 2-fold increase in abstinence among study participants in New Zealand and United Kingdom (20, 21). *Kick Buts* follows principles from the Social Cognitive Theory (26), including motivational messages, behavioral-change support, and pharmacotherapy use (NRT). *Kick Buts*, available in English and Spanish, allowed three levels of interactivity:

1. Pre-scheduled standard messages. Messages were tailored to the participant's name(s) and their selected quit date. The text messaging system delivered these messages according to an algorithm based on four sequential phases of the quitting process: (1) Pre-quit (14 days); (2) Quit-day (1 day); (3) Post-quit Intensive (28 days); and (4) Post-quit Maintenance (8 weeks).
2. Keyword triggered standard messages. These messages consisted of automated responses sent immediately to participants who sent the program one of the following keywords: Alcohol, Crave, Relapse, Slip Up, and Stress. Participants could withdraw from the text messaging program at any time by texting the word Stop.
3. Personalized responses. The text messaging system had the ability to receive free texts (not keywords) from study participants. Trained research staff monitored, triaged, and responded (if needed) to text messages sent by participants. Responses occurred within 48 h of receiving the texts.

### Decision Support

An additional new first text-based stage, Decision Support, was created and integrated with the already existing four stages of Kick Buts (pre-quit, quit-day, post-quit intensive, and post-quit maintenance). The aim of this 10-day stage was to enhance motivation and readiness among Latino smokers who were not ready to quit smoking. The Decision Support stage was informed from two web-based smoking cessation decision-making tools developed for Latinos in the U.S. (27) and Mexico (28). The Decision Support stage helped participants develop their quit plan, identifying “how” (e.g., type of medication) and “when” (e.g., quit date) they wanted to quit smoking. Participants were prompted to set up a quit plan after 3, 6, and 9 days of enrollment by texting READY or LISTO. A trained research staff called participants 24–72 h after texting READY or LISTO to set up a quit date within a 2-week frame and their Nicotine Replacement Therapy (NRT) of preference. If a participant did not text READY or LISTO, they were called when the Decision Support stage ended. After the Decision Support stage, participants received the following progressive stages (pre-quit, quit-day, post-quit intensive, and post-quit maintenance).

### Cultural and Linguistic Adaptation of the Intervention

Using the Cultural Accommodation of Substance Abuse Treatment framework (29), we adapted *Kick Buts* to Latino smokers. The aim of this framework is to enhance components of an intervention to increase congruency with the cultural norms of a specific group and adjust delivery mode (29). In accordance with this model, an initial pilot test with Latino smokers (*n* = 20) was conducted (30). In that study, participants received a Spanish translated version of *Kick Buts*. Results of that study indicated that (1) text messages should address social support from family members, friends, and former smokers, and (2) the translation into Spanish should be improved (30). To address participants' feedback on social support, (1) new keywords (e.g., family and health) and (2) new standard messages elucidating vicarious experiences were developed. To address participants' feedback on the translation, a Community and Communication Advisory Board with members of different Latino nationalities was convened to review the message library and ensure the cultural and linguistic congruency of each message. Readability of final messages was assessed by stage using the Flesch–Kincaid and Fernández Huerta tests for English and Spanish, respectively (31–33). A Flesch–Kincaid score of 100–90, 90–80, or 70–80 indicates that the text readability in English is very easy (5th grade level), easy (6th grade level), or fairly easy (7th grade level), respectively (31, 32). A Fernández Huerta score of 100–90, 90–80, or 70–80 indicates that the text readability is very easy (4th grade level), easy (5th grade level), or quite easy (6th grade level), respectively (33).

### Nicotine Replacement Therapy (NRT)

The use of NRT followed the Clinical Practice Guidelines for Treatment of Tobacco Use (16). NRT (nicotine patches, gum, or lozenges) was offered to eligible participants at no cost. NRT was contraindicated and thus not offered to participants who (1) had a heart attack in the past 2 months, (2) had a stroke in the last 6 months, (3) had an arrhythmia or tachycardia, (4) had uncontrolled hypertension, and (5) were using Warfarin at the time of eligibility (16). At the end of the Decision Support stage, each participant received a 4-week supply of their NRT of preference mailed to their address. Participants were encouraged to start using their NRT on their selected quit-date, which was set at their baseline visit. At the beginning of the second week of the intervention, participants received text message prompts to request an NRT refill to continue treatment. If participants responded to the text prompts indicating interest in NRT, a 4- or 6-week supply was shipped to their home.

### Assessments

All assessments were completed in the language of preference of the participant. The in-person baseline survey collected sociodemographic variables such as gender, age, education level, country of birth, language spoken at home, marital status, and type of health insurance. Smoking-related variables collected included number of cigarettes per day (CPD), time to first cigarette, if they made a quit attempt in the previous year, smoking in the social network, contraindications of NRT, and the smoking self-efficacy questionnaire (SEQ-12). The SEQ-12 is a questionnaire measuring the confidence of smokers (current and former) in their ability to abstain from smoking in high-risk situations (34). The SEQ-12 consists of 12 items, and each item is rated on a 5-point Likert scale (1 = not at all sure, 2 = not very sure, 3 = more or less sure, 4 = fairly sure, and 5 = absolutely sure) (34). SEQ-12 scores range from 12 to 60 with higher scores indicating greater self-efficacy (34).

Two brief surveys were done over the phone after baseline: (1) 24–27 h after the end of the Decision Support stage, and (2) 24–72 h after participants' quit date. The survey at the end of the Decision Support stage collected data on participants' quit date and NRT of preference. The survey after participants' quit date collected data on the receipt of NRT and participants' determination to quit smoking on their quit date (Did you try to quit smoking on your quit date?).

During the 12-week duration of the intervention, we assessed the frequency of text messages that participants sent to the program. Participants' texts were categorized as keywords and free-text response to the counselor.

Twelve weeks after enrollment, a second in-person assessment was conducted by trained research staff. Moreover, saliva samples and exhaled carbon monoxide (CO) were collected to verify cessation status. The survey collected data on self-reported prevalence of smoking abstinence, therapeutic alliance, and self-efficacy. Cotinine-verified 7-day point prevalence abstinence (no cigarettes in the past 7 days) at 12 weeks was the primary outcome. Cotinine verification was conducted using saliva samples with a cutoff point of 15 ng/ml cotinine (35, 36). If the participant was using NRT at the time of the 12-week assessment, exhaled CO (with a cutoff of 6 ppm) was used to verify smoking abstinence. Secondary outcomes were satisfaction with the intervention, therapeutic alliance, and changes in self-efficacy. Satisfaction measures included questions such as “How satisfied are you with the smoking cessation text messaging program?” Therapeutic alliance and self-efficacy were measured using the Working Alliance Inventory—Short Version (WAI-S) (37, 38) and the SEQ-12 tests, respectively. The WAI-S is a questionnaire measuring three key aspects of the therapeutic alliance: (1) agreement on the goals of therapy, (2) agreement on the tasks of therapy, and (3) development of an affective bond (37). The WAI-S consists of 12 items, and each item is rated on a 7-point Likert scale (1 = never, 2 = rarely, 3 = occasionally, 4 = sometimes, 5 = often, 6 = very often, and 7 = always) (37, 38). WAI-S scores range from 12 to 84, with higher scores reflecting a stronger working alliance (37, 38).

### Analysis

Frequencies were calculated for categorical variables. Means and standard deviations were calculated for continuous variables. For the primary analysis on smoking cessation, participants lost to follow-up were considered smokers. The secondary analysis on satisfaction, therapeutic alliance, and self-efficacy were conducted using complete case analysis, in which missing values in the outcome were considered to be missing. Self-efficacy at baseline and follow-up was compared using a paired sample *T*-test to examine differences. Associations between cessation and sample characteristics were analyzed using Chi-square tests. Fisher test was used when cells had expected counts <5.

## Results

The average readability score of final messages in English and Spanish was classified as “very easy”. The reading levels of each text category were “very easy” (50%) and “quite easy” (50%). Scores averaged 80.01 (range 73.41–86.46, 6th−7th grade levels) and 83.39 (range 77.80–90.56, 4th−6th grade levels) using the Flesch–Kincaid and Fernández Huerta tests, respectively. Readability levels were similar in English and Spanish for all stages of the text message library (see Table 1).

**Table 1:** Readability results of text messages.

**Stage**	**Flesch–Kincaid score (English)**			**Fernández Huerta (Spanish)**		
	**Score**	**Level**	**Grade level**	**Score**	**Level**	**Grade level**
Decision support	74.23	Fairly easy	7th grade	78.00	Quite easy	6th grade
Pre-quit	81.00	Easy	6th grade	83.40	Easy	5th grade
Quit-day	80.10	Easy	6th grade	84.58	Easy	5th grade
Post-quit intensive	76.19	Fairly easy	7th grade	80.65	Easy	5th grade
Post-quit maintenance	77.77	Fairly easy	7th grade	82.20	Easy	5th grade
Health	86.46	Easy	6th grade	90.56	Very easy	4th grade
Family	84.75	Easy	6th grade	88.85	Easy	5th grade
Patch	82.09	Easy	6th grade	86.22	Easy	5th grade
Lozenge	76.58	Fairly easy	7th grade	80.86	Easy	5th grade
Gum	73.41	Fairly easy	7th grade	77.80	Quite easy	6th grade
Crave	79.91	Fairly easy	7th grade	80.11	Easy	5th grade
Stress	82.19	Easy	6th grade	84.44	Easy	5th grade
Alcohol	84.30	Easy	6th grade	86.12	Easy	5th grade
Slip up	81.22	Easy	6th grade	83.78	Easy	5th grade
Average	80.01	Easy	6th grade	83.39	Easy	5th grade

**Figure 1:**
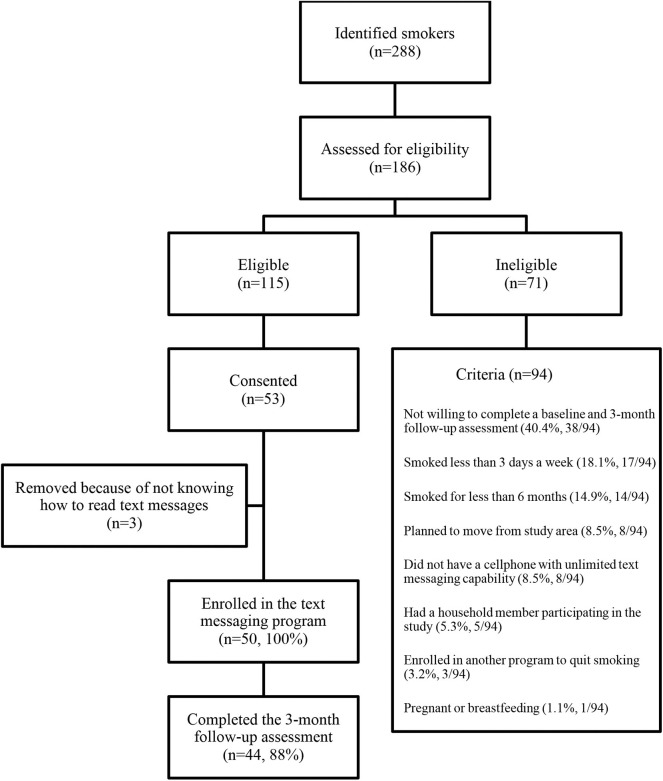
Study design flow diagram.

A total of 288 Latino smokers were identified. Among these, 186 were contacted and assessed for study eligibility; 115 were eligible to participate in the study. Overall, 53 Latino smokers consented to participate the study and completed the baseline assessment. Three smokers were removed from the study because they did not know how to send/receive text messages, resulting in 50 individuals enrolled in the study (Figure 1).

At baseline, participants' mean age was 44.8 years old (SD 9.80), 66% of the participants were male, 64% were married or cohabitating, 60% had completed high school education or greater, and the majority (78%) had no health insurance. Most of the participants (82%) were born in Mexico and 60% only spoke Spanish at home. Most participants (68%) were light smokers (1–10 CPD) and tried to quit in the past year (70%). Half (52%) of the participants smoked their first cigarette more than 60 min after waking up. A third of the sample (32%) reported that none of their five closest family or friends smoked. The mean SEQ-12 score was 32.18 (SD 10.29), which is considered to be in the low to moderate range of self-efficacy for quitting smoking (see Table 2). All participants were eligible to use NRT. Nearly all participants (94%) chose the intervention in Spanish.

**Table 2:** Baseline characteristics of participants (*n* = 50).

**Characteristics**	***n* (%)**
Age, Mean (SD)	44.8 (9.8)
Gender	
Female	17 (34%)
Male	33 (66%)
Marital status	
Married/cohabitating	32 (64%)
Single	3 (6%)
Divorced/separated/widowed	15 (30%)
Education level	
Less than high school graduate	20 (40%)
High school graduate	22 (44%)
Technical school	5 (10%)
College graduate	3 (6%)
Health insurance coverage	
No health coverage	39 (78%)
Private	8 (16%)
Public (Medicare, Medicaid)	3 (6%)
Country of birth	
Mexico	41 (82%)
Guatemala	3 (6%)
United States	2 (4%)
Honduras	2 (4%)
Chile	1 (2%)
Nicaragua	1 (2%)
Language spoken at home	
Only Spanish	30 (60%)
More Spanish than English	15 (30%)
Spanish and English equally	4 (8%)
More English than Spanish	1 (2%)
Only English	0
Smoking pattern	
Non-daily	2 (4%)
Daily, 1–10 CPD	34 (68%)
Daily, 11–20 CPD	13 (26%)
Daily, 21 or more CPD	1 (2%)
Time to first cigarette after waking up	
>60 min	26 (52%)
31–60 min	8 (16%)
6–30 min	5 (10%)
≤ 5 min	11 (22%)
Attempted to quit smoking in previous year	
Yes	35 (70%)
No	15 (30%)
Number of 5 closest friends/family who smoke	
0	16 (32%)
1	12 (24%)
2	6 (12%)
3	8 (16%)
4	3 (6%)
5	5 (10%)
Self-efficacy (SEQ-12), Mean (SD)	32.18 (10.29)

*CPD, Cigarettes per day; SEQ-12, Self-efficacy questionnaire-12*.

All participants were reached at the end of the decision support stage and set up a quit date. All participants requested the first order of NRT. Thirty-six participants (72%) requested nicotine patches, 10 participants (20%) requested nicotine gum, and four participants (8%) requested nicotine lozenges. Forty-two participants were reached after their quit day (84% retention rate). All participants reported receiving their first order of NRT. A total of 95.23% participants (40/42) reported doing an NRT-assisted attempt on their quit day.

During the 12-week period of the intervention, levels of text messaging interactivity varied among participants: 11 participants (22%) had a low interaction with the program (1–9 text messages), 18 (36%) had a medium interaction (10–49 text messages), 11 (22%) had a high interaction (50–99 text messages), and 10 (20%) had a very high interaction (≥100 text messages). All participants interacted with the program and had on average of 39.78 (SD 82.70) interactions. Of the 1,989 messages that participants sent to the program, 280 (14.07%) used keywords. Only 3 participants (6%) discontinued the program prior to completion by texting Stop. Of the 50 participants, 33 (66%) requested a refill of NRT via a text message.

At 12 weeks, 15 participants (30%) were biochemically verified abstinent (Table 3). The follow-up rate at 12 weeks was 88%. Most participants (90.90%, 40/44) reported being very satisfied or extremely satisfied with the intervention. Working alliance mean values was 79.2 (SD 9.04), which is considered in the high range of therapeutic alliance in quitting smoking. Self-efficacy mean scores significantly increased (*n* = 41) from 33.98 (SD 10.36) at baseline to 40.05 (SD 17.65) at follow-up (*p* = 0.04). Cessation at week 12 by gender, education level, health insurance cover, and smoking pattern is shown in Table 4. None of these characteristics was significantly associated with cessation.

**Table 3:** 12 weeks follow-up outcomes.

**Outcome**	**Statistics**
Biochemically verified abstinence (*n* = 50), *n* (%)	15 (30%)
Satisfaction (*n* = 44), *n* (%)	
Extremely satisfied	16 (36.4%)
Very satisfied	24 (54.6%)
Somewhat satisfied	4 (9.0%)
Working Alliance (*n* = 44), Mean (SD)	79.2 (9.04)
Self-efficacy (SEQ-12) (*n* = 41), Mean (SD)	40.05 (17.65)

*SEQ-12, Self-efficacy questionnaire-12*.

**Table 4:** Associations between smoking cessation and sample characteristics.

**Characteristics**	**Total (*n* = 50)**	**Quit smoking (*n* = 15)**	***p*-value**
Gender			
Female	17	4 (23.5%)	0.533^a^
Male	33	11 (33.3%)	
Education level			
Less than high school graduate	20	8 (40.0%)	0.228^b^
High school graduate	22	6 (27.3%)	
Technical school	5	1 (20.0%)	
College graduate	3	0 (0%)	
Health insurance coverage			
No	39	13 (33.3%)	0.468^c^
Private	8	2 (25.0%)	
Public (Medicare, Medicaid)	3	0 (0%)	
Smoking pattern			
Non-daily	2	1 (50%)	0.507^d^
Daily, 1–10 CPD	34	11 (32.4%)	
Daily, 11–20 CPD	13	3 (23.6%)	
Daily, 21 or more CPD	1	0 (0%)	

*CPD, Cigarettes per day*.

a *Fisher's exact test*.

b *Fisher's exact test. The Education Level variable was dichotomized into “Less than high school graduate” vs. “High school graduate or higher education.” The p-value compares these two groups*.

c *Fisher's exact test. The Health Insurance Coverage variable was dichotomized into “No insurance” vs. “Private or public insurance.” The p-value compares these two groups*.

d *Fisher's exact test. The Smoking Pattern variable was dichotomized into “10 or less CPD” vs. “11 or more CPD.” The p-value compares these two groups*.

## Discussion

To our knowledge, this is the first culturally- and linguistically adapted smoking cessation text messaging intervention for Latino smokers. In this study, participants expressed high levels of interest in using NRT combined with high engagement in the text messaging program, with 100% requesting NRT at baseline. Two-thirds of participants (66%) requested additional NRT via the text messaging program, suggesting that participants completed at least a 4-week course of medication. Finally, the 30% smoking cessation rate seen at week 12 (end of treatment) is consistent with end-of-treatment cessation rates seen in trials that combine NRT with substantial in-person counseling (39). This level of interest and encouraging abstinence with use of NRT is particularly promising given few clinical trials have included pharmacotherapy for Latino smokers (14, 40–42), and older studies have suggested limited interest in use of pharmacotherapy generally and NRT specifically among Latinos (7). Current findings suggest high interest and utilization.

### Implications for Future Research

This work demonstrates that it is feasible to recruit Latino smokers, a traditionally hard-to-reach group, into a smoking cessation study. As suggested by Cupertino et al., trained *Promotores de Salud* were a key resources for recruiting this underserved population (43). Future studies can build upon this community-based approach to conduct tobacco control research among Latinos.

We carefully considered the eligibility criteria with the goal of maximizing participation of Latino smokers. Latinos are among the racial/ethnic minority groups with the highest rates of non-daily smoking (44). In the present study, individuals who smoke more than 3 days a week were eligible to participate. Two non-daily smokers were eligible and enrolled in the study (4% of the total sample). However, smoking <3 days a week was the second most common reason for ineligibility (18.08%, 17/94). Given this relatively high number, and that smoking cessation interventions are almost non-existent for non-daily smokers, it is imperative to design, develop, and implement interventions for this particular group.

Moreover, three subjects were removed from the study after consenting because of not knowing how to read text messages. This decision was based on the rationale that to test the impact of the text messaging intervention in smoking cessation, participants need to know how to read the messages. Future studies that aim to test the impact of text messaging interventions, should include an eligibility criteria in which participants must know how to read text messages.

Four studies have assessed participant interactivity in smoking cessation text messaging programs (30, 45–47). Interestingly, participants in our study interacted at higher levels (34.6 text messages per participant throughout the 12-week period) in this study. Abroms et al. found that among two mostly non-Latino samples of smokers, those with at least one interaction with the program sent an average of 11.8 and 28.4 text messages during a 6-month period (45, 46). Of importance, the interactivity in these studies was keyword-based (45, 46). Similar to the studies conducted by Cartujano-Barrera et al. (30) and Cupertino et al. (47), participants in this study mostly sent their own, self-composed text messages rather than relying on keywords for a program response. This finding reinforces the hypothesis that text messaging interactivity via keywords may not be sufficient for smoking cessation among Latinos. The text messaging program shows promise to be a low-cost alternative to in-person or telephone counseling for smoking cessation. Moreover, additional strategies can be implemented to lessen the cost of having trained staff responding to participants' text messages, as took place in this study. Messages that participants sent to the program can also inform the development of a text messaging responses codebook, in which responses would be retrieved and sent automatically, thus reducing the need for trained staff responding to participants' messages.

This study provided preliminary evidence that a smoking cessation text messaging intervention significantly increases self-efficacy. As results from myriad cross-sectional and prospective correlational studies show that self-efficacy is a consistent predictor of smoking abstinence (48), the role of text messaging interventions to increase self-efficacy should be further evaluated. Furthermore, participants in this study reported a very strong therapeutic alliance at end-of-treatment. Greater working alliance predicts early engagement and positive outcomes in many but not all studies on counseling for substance use disorder (49). The role of therapeutic alliance has received some attention in the smoking cessation literature (50, 51). However, more research is needed to assess this counseling mechanism, especially in the context of mobile technologies. Future studies should assess therapeutic alliance and self-efficacy as mediators of the presumed treatment effect on smoking abstinence among Latino smokers using smoking cessation technologies.

### Limitations

This study had a number of limitations. This was a pilot study with a small sample size and no comparison group was available. Thus, assessing the efficacy of this intervention was not possible. Furthermore, follow-up was limited to a single assessment at the end of treatment at 12-week, and does not include assessment of sustained behavior change after treatment. Despite these limitations, the study suggests that this intervention holds promise as an effective smoking cessation program for Latino smokers, and further testing in a randomized clinical trial is warranted.

We recognize while Latinos come from a variety of countries, most Latinos in this study were Mexican. It remains unknown whether these preliminary results can be generalized to the entire Latino population, especially since smoking rates vary significantly by country of origin. For instance, a U.S. population-based longitudinal study reported that current smoking rates was highest among Puerto Ricans (men 35.0% and women 32.6%) and Cubans (men 31.3% and women 21.9%), with significantly high smoking intensity measured by pack-years and cigarettes per day among Cubans (52). Dominicans had the lowest smoking prevalence (men 11.0% and women 11.7%). Latinos of other national backgrounds had smoking rates between these groups, and typically higher among men compared to women. Moreover, Puerto Ricans and Cubans were the least likely to report successful quit attempts compared to other Latino subgroups (53). Finally, participants in this study appeared to be low-acculturated given that the majority of participants spoke only Spanish. Future research should consider assessing the efficacy of this intervention among Latinos with varied acculturation levels.

## Conclusion

A culturally- and linguistically-adapted smoking cessation text messaging intervention for Latinos was well-accepted by participants, generated high satisfaction and frequent interactivity, significantly increased self-efficacy, produced high therapeutic alliance, and resulted in noteworthy cessation rates at the end of treatment. Moreover, the intervention offers a promising strategy to increase the use and adherence of Nicotine Replacement Therapy. Additional testing in a formal randomized clinical trial is warranted to identify the effectiveness of the intervention and to determine individual characteristics related to treatment response.

## Data Availability Statement

The datasets generated for this study are available on request to the corresponding author.

## Ethics Statement

The study was reviewed and approved by University of Kansas Medical Center Institutional Review Board. Participants provided their written informed consent to participate in this study.

## Author Contributions

AC, LS, and EE designed the study. FC-B, MR, and JP-P implemented the study. FC-B, EA-C, MR, JP-P, and FD analyzed the data. FC-B, LS, EA-C, MR, JP-P, PV, FD, DC, EE, and AC wrote and revised multiple versions of the manuscript and including the final version. All authors contributed to the article and approved the submitted version.

## Conflict of Interest

The authors declare that the research was conducted in the absence of any commercial or financial relationships that could be construed as a potential conflict of interest.
